# Genetic Variants of Human Granzyme B Predict Transplant Outcomes after HLA Matched Unrelated Bone Marrow Transplantation for Myeloid Malignancies

**DOI:** 10.1371/journal.pone.0023827

**Published:** 2011-08-23

**Authors:** Luis J. Espinoza, Akiyoshi Takami, Katsuya Nakata, Kayoko Yamada, Makoto Onizuka, Takakazu Kawase, Hiroshi Sao, Hideki Akiyama, Koichi Miyamura, Shinichiro Okamoto, Masami Inoue, Takahiro Fukuda, Yasuo Morishima, Yoshihisa Kodera, Shinji Nakao, for the Japan Marrow Donor Program

**Affiliations:** 1 Department of Hematology and Oncology, Kanazawa University Hospital, Kanazawa, Japan; 2 Department of Hematology and Oncology, Tokai University School of Medicine, Isehara, Japan; 3 Division of Epidemiology, Aichi Cancer Center Hospital, Nagoya, Japan; 4 Department of Hematology and Cell Therapy, Aichi Cancer Center Hospital, Nagoya, Japan; 5 Department of Hematology, Meitetsu Hospital, Nagoya, Japan; 6 Department of Internal Medicine, Ebara Hospital, Tokyo, Japan; 7 Department of Hematology, Japanese Red Cross Nagoya First Hospital, Nagoya, Japan; 8 Division of Hematology, Department of Medicine, Keio University School of Medicine, Tokyo, Japan; 9 Department of Hematology and Oncology, Osaka Medical Center and Research Institute for Maternal and Child Health, Osaka, Japan; 10 Hematopoietic Stem Cell Transplantation Unit, National Cancer Center Hospital, Tokyo, Japan; 11 Department of Promotion for Blood and Marrow Transplantation, Aichi Medical University, Nagoya, Japan; Instituto Nacional de Câncer, Brazil

## Abstract

Serine protease granzyme B plays important roles in infections, autoimmunity, transplant rejection, and antitumor immunity. A triple-mutated granzyme B variant that encodes three amino substitutions (Q48R, P88A, and Y245H) has been reported to have altered biological functions. In the polymorphism rs8192917 (2364A>G), the A and G alleles represent wild type QPY and RAH mutant variants, respectively. In this study, we analyzed the impact of granzyme B polymorphisms on transplant outcomes in recipients undergoing unrelated HLA-fully matched T-cell-replete bone marrow transplantation (BMT) through the Japan Donor Marrow Program. The granzyme B genotypes were retrospectively analyzed in a cohort of 613 pairs of recipients with hematological malignancies and their unrelated donors. In patients with myeloid malignancies consisting of acute myeloid leukemia and myelodysplastic syndrome, the donor G/G or A/G genotype was associated with improved overall survival (OS; adjusted hazard ratio [HR], 0.60; 95% confidence interval [CI], 0.41–0.89; *P* = 0.01) as well as transplant related mortality (TRM; adjusted HR, 0.48; 95% CI, 0.27–0.86, *P* = 0.01). The recipient G/G or A/G genotype was associated with a better OS (adjusted HR, 0.68; 95% CI, 0.47–0.99; *P* = 0.05) and a trend toward a reduced TRM (adjusted HR, 0.61; 95% CI, 0.35–1.06; *P* = 0.08). Granzyme B polymorphism did not have any effect on the transplant outcomes in patients with lymphoid malignancies consisting of acute lymphoid leukemia and malignant lymphoma. These data suggest that there is an association between the granzyme B genotype and better clinical outcomes in patients with myeloid malignancies after unrelated BMT.

## Introduction

Hematopoietic stem cell transplantation (HSCT) represents the only potentially curative option for many malignant conditions. Although substantial improvements in the supportive care of transplanted patients have been achieved in recent years, the profound compromise in the immune system associated with HSCT constitutes a significant risk for life threatening complications including GVHD, severe infections and disease relapse.[1] HLA matching represents the major genetic determinant in clinical outcomes after allogeneic HSCT, however, several studies have suggested that non-HLA genes associated with immune functions are also involved in determining the clinical outcome.[2] Single nucleotide polymorphisms (SNPs) in genes involved in the immune response to infections and inflammatory reactions have been identified as additional predictive markers of clinical outcomes in HSCT.[3], [4], [5], [6], [7], [8], [9], [10], [11], [12], [13], [14]

Following HSCT, cytotoxic T lymphocytes (CTLs) and natural killer (NK) cells, mainly derived from the donor, constitute the most important effector cells that eliminate allogeneic cells, including malignant cells, virus-infected cells and healthy cells. The destruction of the target cells occurs by at least one of the three defined mechanisms: TNF-α release, the Fas/Fas ligand interaction, and the granzyme/perforin pathway.[15] The later has been postulated as being the predominant mechanism for immune-mediated apoptosis of allogeneic cells.[16], [17]

Granzyme B, the most abundant serine protease stored in secretory granules of CTLs and NK cells, is released upon target cell recognition, then specifically enters into the target cell cytoplasm via perforin, finally leading to target cell lysis.[15] Although the induction of target cell death by its pro-apoptotic properties has been considered the central function of granzyme B, growing evidence indicates that this protease also possesses additional non-death-related functions. These non-classical or extracellular functions are perforin-independent mechanisms and include immunosuppression, receptor cleavage, and cytokine-like effects.[15], [18], [19] Initially believed to be expressed exclusively by NK cells and CTLs, recent reports have shown that granzyme B can be expressed by various additional cell types, such as mast cells, neutrophils, dendritic cells (DCs), B cells, keratinocytes, chondrocytes, and vascular smooth muscle cells.[20], [21], [22], [23], [24]

Granzyme B is involved in the pathophysiology of viral and bacterial infections, solid organ rejection, autoimmune diseases, and antitumor immunity.[25], [26], [27], [28], [29], [30] In the granzyme B gene, a triple-mutated allele (Q48R, P88A, and Y245H) in strong linkage disequilibrium is found in European, African, and Asian populations, including the Japanese population, at an allelic frequency of 25–30%.[31], [32] The biological and functional relevance of the RAH mutant granzyme B, however, still remains controversial. Although it was reported that the RAH variant was incapable of inducing apoptosis,[31] and γδT cells derived from donors possessing the RAH variant had impaired cytotoxicity against target cells;[33] other studies have reported that RAH mutant granzyme B displays normal proteolytic and cytotoxic properties[34] and the cytotoxicic activity of T lymphocytes did not differ among donors with QPY or RAH genotypes.[32]

In this study, we hypothesized that a defect of inducing apoptosis in mutant granzyme B could influence the clinical outcomes of HSCT. To test this hypothesis, we investigated the influence of the QPY/RAH variants on the clinical outcomes after HSCT. Because these variants are in clear linkage disequilibrium, the study was focused on genotyping the polymorphism rs8192917 (2364A>G) in the granzyme B gene, which results in Q48R variants, and analyzed its impact on the clinical outcomes of patients undergoing allogeneic bone marrow transplantation (BMT) using an HLA allele-matched unrelated donor. The data herein show that the donor G/G or A/G allele, which represents mutant granzyme B, is associated with a significantly improved overall survival (OS) and reduced transplant-related mortality (TRM) in patients with myeloid malignancies.

## Methods

### Patients

Granzyme B genotyping was performed on 613 recipients with hematological malignancies and their unrelated donors who underwent BMT through the Japan Marrow Donor Program (JMDP) with T-cell-replete marrow from HLA-A, -B, -C, -DRB1, -DQB1, and -DPB1 allele-matched donors between January 1993 and December 2007. The HLA genotypes of patients and donors were determined by the Luminex microbead method, as described previously (Luminex 100 System; Luminex, Austin, TX).[35], [36] Although the Luminex microbead method does not provide unambiguous HLA 4-digit typing for all genotypes, the JMDP has confirmed that this method can identify all HLA alleles with >0.1% frequency among the Japanese population.[37]

None of the present patients had a history of any prior transplantation. The final clinical survey of these patients was completed by November 1, 2008. The diagnoses were acute myeloid leukemia (AML) in 240 (39%), acute lymphoblastic leukemia (ALL) in 170 (28%), myelodysplastic syndrome (MDS) in 113 (18%), and malignant lymphoma (ML) in 90 (15%; **Tables 1** **and** **2**). The recipients were defined as having standard risk disease if they had AML or ALL in the first complete remission, ML in any complete remission, or MDS. All others were designated as having high-risk disease. The myeloid malignancies include AML and MDS, and the lymphoid malignancies included ALL and ML. Cyclosporine- or tacrolimus-based regimens were used in all patients for GVHD prophylaxis, and anti-T cell therapy, such as anti-thymocyte globulin and *ex vivo* T cell depletion were not in any of the patients. All patients and donors gave their written informed consent at the time of transplantation to participate in molecular studies of this nature according to the declaration of Helsinki. This project was approved by the Institutional Review Board of Kanazawa University Graduate School of Medicine and the JMDP.

**Table 1:** Donor and recipient characteristics (first part).

Variable	No.	Ratio
No. of cases	613	
Recipient age, years		
Median	36	
Range	1–70	
Donor age, years		
Median	34	
Range	20–57	
Year of transplant		
Median	2002	
Range	1993–2007	
Recipient Granzyme B genotype		
G/G	30	5%
A/G	202	33%
A/A	381	62%
Donor Granzyme B genotype		
G/G	27	4%
A/G	194	32%
A/A	392	64%
Recipient sex		
Male	383	62%
Female	230	38%
Donor sex		
Male	402	66%
Female	210	34%
Missing	1	0%
Donor/recipient sex		
Sex matched	409	67%
Female/male	92	15%
Male/female	111	18%
Missing	1	0%

**Table 2:** Donor and recipient characteristics (second part).

Variable	No.	Ratio
Disease		
Acute myeloid leukemia	240	39%
Myelodysplastic syndrome	113	18%
Acute lymphoblastic leukemia	170	28%
Malignant lymphoma	90	15%
Disease stage		
Standard risk	357	58%
High risk	256	42%
ABO matching		
Major or/and minor mismatch	246	40%
Major mismatch	136	22%
Minor mismatch	126	21%
Bidirectional	18	3%
Missing	7	1%
Conditioning regimen		
Myeloablative	499	81%
Reduced intensity	114	19%
With total body irradiation	472	77%
Pretransplant CMV serostatus		
CMV positive recipient	440	72%
Missing	70	11%
GVHD prophylaxis		
With cyclosporine	296	48%
With tacrolimus	314	51%
Missing	3	0%
TNC, × 10^8^ per kg		
Median	4.9	
Range	0.1–79.1	

Abbreviations: TNC: total nucleated cell count harvested.

### Granzyme B genotyping

Genotyping of granzyme B was performed using the TaqMan-Allelic discrimination method in a StepOne Plus Real Time PCR system (Applied Biosystems, Foster City, CA, USA), and the results were analyzed using the Allelic Discrimination software program (Applied Biosystems). The genotyping assay was conducted in 96-well PCR plates. The amplification reaction contained template DNA, TaqMan universal master mix and the specific probe designed for SNP rs8192917 (2364A>G) of granzyme B (product No. C_2815152_20 ; Applied Biosystems).

### Data management and statistical analysis

The data were collected by the JMDP using a standardized report form. Follow-up reports were submitted at 100 days, 1 year and annually after transplantation. The pre-transplant cytomegalovirus (CMV) serostatus was routinely tested for only patients, but not the donors. Engraftment was confirmed by an absolute neutrophil count of more than 0.5×10^9^/L for at least 3 consecutive days. On collecting data, acute and chronic GVHD were diagnosed and graded using the previous criteria[38], [39] and data using updated criteria for assessment of GVHD[40], [41] were not available in our cohort. The OS was defined as the number of days from transplantation to death from any cause. Disease relapse was defined as the number of days from transplantation to disease relapse. TRM was defined as death without relapse. Any patients who were alive at the last-follow-up date were censored. The data about causative microbes of infections and postmortem changes in cause of death, as well as the data on supportive care, including prophylaxis for infections and therapy for GVHD, which were given on an institutional basis, were not available for this cohort.

The analysis was performed using the Excel 2007 (Microsoft Corp, Redmond, WA, USA) and modified R (The R Foundation for Statistical Computing, Perugia, Italy) software programs.[42], [43] The probability of overall survival (OS) was calculated using the Kaplan-Meier method and compared using the log-rank test. The probabilities of TRM, disease relapse, acute GVHD, chronic GVHD, and engraftment were compared using the Grey test[44] and analyzed using the cumulative incidence analysis,[42] considering relapse, death without disease relapse, death without acute GVHD, death without chronic GVHD, and death without engraftment as respective competing risks. The variables were recipient age at time of transplantation, sex, pretransplant CMV serostatus, disease characteristics (disease type, disease lineage and disease risk at transplantation), donor characteristics (age, sex, sex compatibility, and ABO compatibility), transplant characteristics (conventional or reduced-intensity conditioning,[45] total body irradiation-containing regimen, tacrolimus versus cyclosporine, and total nucleated cell count harvested per recipient weight [TNC]), and the year of transplantation. The median was used as the cutoff point for continuous variables. The chi-square test and Mann-Whitney U test were used to compare two groups. The Hardy-Weinberg equilibrium for the granzyme B gene polymorphism was tested using the Haploview software program.[6]

Multivariate Cox models were used to evaluate the hazard ratio associated with the granzyme B polymorphism. Covariates found to be P≤0.10 in the univariate analyses were used to adjust the hazard ratio. The covariates were selected according to myeloid and lympoid malignancies.

For both the univariate and multivariate analyses, P values were two sided, and outcomes were considered to be significant for P≤0.05.

## Results

### The frequencies of the granzyme B genotypes

Granzyme B gene polymorphism was analyzed in 613 unrelated bone marrow donor-transplant recipient pairs (**Tables 1** **and** **2**). The genotype frequencies of G/G, A/G and A/A were 5%, 33% and 62% in recipients and 4%, 32% and 64% in donors. These were similar to HapMap data in the Japanese (9%, 29% and 62%, respectively) and European (5%, 35% and 60%, respectively) populations, and thus were in accord with the Hardy-Weinberg equilibrium (*P* = 0.79).

### Transplant outcome according to the granzyme B genotype

The median follow-up duration in the cohort was 55 months among the survivors (range 4 to 168 months), and 191 recipients (31%) had relapsed or progressed, and 309 (50%) had died. Eighteen patients (3%) died before engraftment. The donor and recipient granzyme B genotype did not significantly influence the cumulative incidence of engraftment (data not shown).

**Figure 1:**
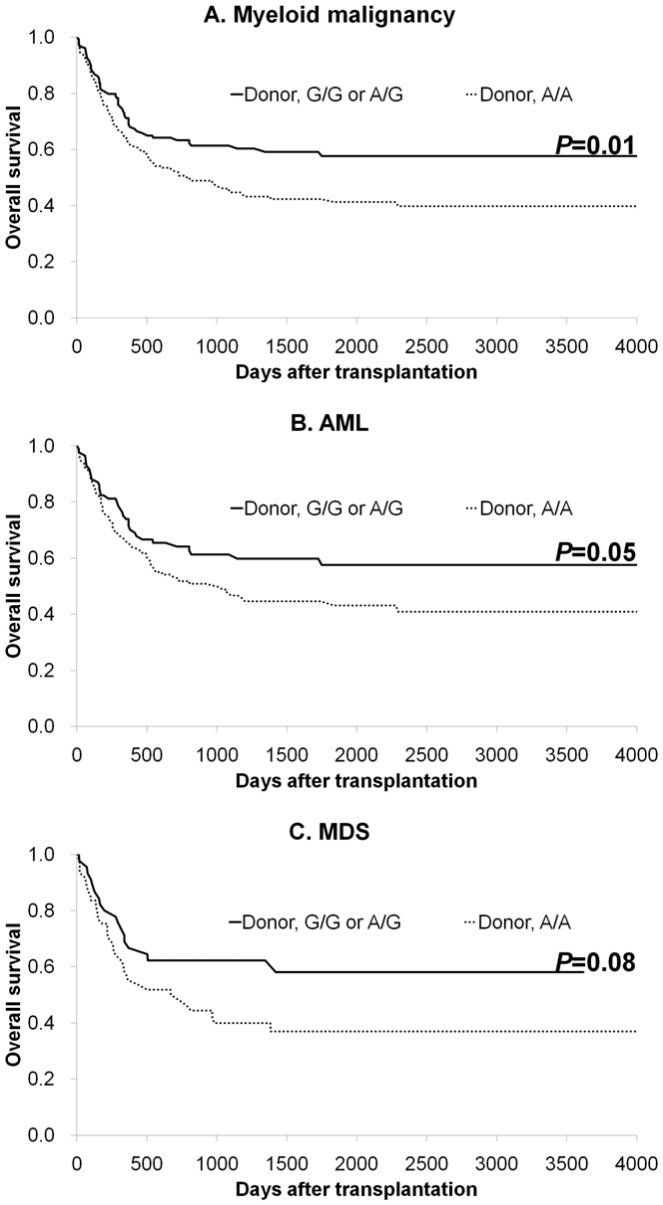
The Kaplan-Meier analysis of OS after BMT according to the donor granzyme B genotype in patients with myeloid malignancies (A), AML (B), and MDS (C).

The transplant outcomes according to the granzyme B genotype are summarized in **Table 3**. Patients with myeloid malignancies, which included AML and MDS, who received transplants from donors with the G/G or A/G genotype had a significantly better 5-year OS (58% vs. 42%, *P* = 0.01; **Fig. 1A**) and a trend toward lower 5-year relapse rate (27% vs. 36%, *P* = 0.09) than those receiving transplants from donors with the A/A genotype. No difference was noted in the TRM, II-IV acute GVHD, or chronic GVHD in relation to the donors' polymorphism status. A comparison between the donor G/G and A/G genotypes showed no significant difference in OS (71% vs. 56%, *P* = 0.36), TRM (6% vs. 23%, *P* = 0.36), or the relapse rate (30% vs. 26%, *P* = 0.65). When patients with AML and MDS were separately analyzed, the donor G/G or A/G genotypes remained statistically significant for a better OS in AML patients (58% vs. 45%; **Fig. 1B**), and had a tendency to be related to a better OS in MDS patients (58% vs. 37%; **Fig. 1C**). In patients with lymphoid malignancies consisting of ALL and ML, the donor granzyme B genotype had no significant effects on transplant outcomes (**Table 3**). This was true even when ALL and ML were separately analyzed (data not shown).

**Table 3:** The results of the univariate analysis of the association of the Granzyme B genotype with clinical outcomes after transplantation.

Variable	No.	5-year OS	*P*	5-year TRM	*P*	5-year relapse	*P*	II-IV acute GVHD	*P*	Chronic GVHD	*P*
Myeloid malignancy	353										
Recipient Granzyme B genotype											
A/G or G/G	139	52%	0.13	21%	0.17	33%	0.71	25%	0.23	40%	0.14
A/A	214	46%		26%		33%		31%		49%	
Donor Granzyme B genotype											
A/G or G/G	141	58%	0.01	20%	0.21	27%	0.10	28%	0.72	47%	0.79
A/A	212	42%		26%		37%		30%		45%	
Lymphoid malignancy	260										
Recipient Granzyme B genotype											
A/G or G/G	93	48%	0.14	24%	0.26	35%	0.97	35%	0.66	31%	0.44
A/A	167	43%		26%		34%		33%		36%	
Donor Granzyme B genotype											
A/G or G/G	80	43%	0.93	29%	0.60	33%	0.78	34%	0.88	32%	0.49
A/A	180	46%		24%		35%		34%		36%	

Abbreviations: OS, overall survival; TRM, Transplant-related mortality.

### Multivariate analysis

All of the factors found to be significant in the univariate analyses were included in the model. In patients with myeloid malignancies, the G/G or A/G genotype in donors were statistically significant in the multivariate analyses for better OS (adjusted hazard ratio [HR], 0.60; 95% confidence interval [CI], 0.41–0.89; *P* = 0.01; **Table 4**) and TRM (adjusted HR, 0.45; 95% CI, 0.25–0.80; *P* = 0.01) when adjusted for the other factors in the models. Despite not evident in the univariate analysis, the multivariate analysis revealed the donor granzyme B G/G or AG genotype was associated with lower incidence of chronic GVHD (adjusted HR, 0.61; 95% CI, 0.37–0.99; *P* = 0.05; **Table 5**). In the independent analyses for AML patients and MDS patients, beneficial effects on OS by the donor G/G or A/G genotype were also found, which was close to being significant in both the AML patients (adjusted HR, 0.68; 95% CI, 0.42–1.09; *P* = 0.10) and the MDS patients (adjusted HR, 0.61; 95% CI, 0.35–1.08; *P* = 0.09). In addition, the recipient G/G or A/G genotype was associated with a significantly better OS (adjusted HR, 0.68; 95% CI, 0.47–0.99; *P* = 0.05) and a trend toward a reduced TRM (adjusted HR, 0.61; 95% CI, 0.35–1.06; *P* = 0.08). The difference between the donor G/G and A/G genotype did not reach statistical significance in relation to transplant outcomes (data not shown). The granzyme B genotype did not significantly influence the transplant outcomes in patients with lymphoid malignancies.

**Table 4:** The results of a multivariate analysis of the association of the Granzyme B genotype with the clinical outcomes after transplantation.

Variable	OS			TRM			Relapse		
	Adjusted HR	95% CI	*P*	Adjusted HR	95% CI	*P*	Adjusted HR	95% CI	*P*
Myeloid malignancy									
Recipient Granzyme B genotype, G/G or A/G	0.68	0.47–0.99	0.05	0.61	0.35–1.06	0.08	0.99	0.65–1.51	0.97
Donor Granzyme B genotype, G/G or A/G	0.60	0.41–0.89	0.01	0.45	0.25–0.80	0.01	0.75	0.48–1.15	0.19
Lymphoid malignancy									
Recipient Granzyme B genotype, G/G or A/G	0.99	0.60–1.57	0.96	0.93	0.44–1.96	0.84	1.40	0.84–2.34	0.20
Donor Granzyme B genotype, G/G or A/G	0.72	0.43–1.28	0.23	0.84	0.32–2.22	0.72	0.87	0.49–1.56	0.65

**Table 5:** The results of a multivariate analysis of the association of the Granzyme B genotype with GVHD after transplantation.

	II-IV acute GVHD			Chronic GVHD		
Variable	Adjusted HR	95% CI	*P*	Adjusted HR	95% CI	*P*
Myeloid malignancy						
Recipient Granzyme B genotype, G/G or A/G	0.78	0.51–1.19	0.24	0.83	0.53–1.31	0.42
Donor Granzyme B genotype, G/G or A/G	0.94	0.62–1.43	0.76	0.61	0.37–0.99	0.05
Lymphoid malignancy						
Recipient Granzyme B genotype, G/G or A/G	0.90	0.55–1.45	0.69	0.90	0.54–1.50	0.69
Donor Granzyme B genotype, G/G or A/G	1.07	0.65–1.76	0.79	1.13	0.68–1.89	0.64

## Discussion

The current study showed that the granzyme B G/G or A/G genotype at rs8192917 (2364A>G) in the donor side representing the triple variant RAH granzyme B was associated with a significantly better OS and TRM compared to the granzyme A/A genotype, corresponding to wild type QPY granzyme B, for patients with myeloid malignancies receiving HLA-matched unrelated BMT through the JMDP. The G/G or A/G genotypes in the recipient also significantly improved the OS, as well the TRM, although to a lesser extent. This is the first report to show that the granzyme B polymorphism affects transplant outcomes.

The beneficial effects of the G/G or A/G genotype were absent in patients with lymphoid malignancies, irrespective of whether it was ALL or ML. A possible explanation for this may be that ALL and ML cells express the apoptosis inhibitor Bcl-2[46] and the endogenous inhibitor of granzyme B, proteinase inhibitor 9 (PI-9).[47], [48], [49], [50] The expression of these two factors by malignant lymphoid cells may protect them from granzyme B-induced apoptosis and proteolysis[46], [48] and might thus negate the differential effects of the different granzyme B genotypes.

Based on the traditional view that the triple-mutated granzyme B has an impaired pro-apoptotic function, it was expected that the presence of the RAH variant would predict an adverse clinical outcomes after HSCT, namely poor survival or an increased relapse rate. The results presented here, however, do not support that assumption. The mechanisms by which the mutant granzyme B genotype improved transplant outcomes remain unclear. This may be due, in part, because the reports on the biochemical and physiological properties of the triple variant RAH granzyme B are still controversial.

Although an initial study[31] reported that RAH granzyme B was unable to induce apoptosis in tumor cell lines, it was later reported by others that RAH granzyme B retains its pro-apoptotic activity.[34] In addition to the classical role of granzyme B in mediating apoptosis within target cells by NK cells and CTLs, increasing evidence shows that extracellular granzyme B also has alternative functions, including extracellular matrix remodeling, immunosuppressive and cytokine-like effects.[18], [19], [24], [51], [52] A recent report[19] showed that human DCs abundantly secrete granzyme B, which can suppress T-cell expansion. Another report revealed a pivotal function of granzyme B in immunosuppression directed by regulatory T cells, leading to promotion of tumor escape.[52] In addition, extracellular granzyme B potentially induces apoptosis in various organs and tissues, thus leading to chronic inflammatory, autoimmune, and degenerative diseases.[18], [24] In line with these observations, it is plausible that in patients receiving HSCT, extracellular granzyme B could contribute to significant effects, such as modulation of T-cell functions and organ damage, because high serum levels of extracellular granzyme B have been reported in HSCT recipients.[29]

Based on the results presented herein, it may therefore be reasonable to hypothesize that the granzyme B variants have differential biochemical properties whose biological consequences are more relevant on the non-classical functions exerted by the extracellular granzyme B. The analysis of patient serum may offer useful information on this issue, although these samples were not available for the present study. The fact that functional granzyme B is also secreted by nonhematopoietic cells, including keratinocytes, chondrocytes, and smooth muscle cells[18], [53] may explain the findings that granzyme B variants in the recipient side, in addition to that in the donor side, had an impact on the transplant outcomes. Furthermore, this finding supports the view that the presence of the triple-mutated Granzyme B is indeed responsible for the beneficial effect in HSCT for myeloid malignancies.

The effects of the granzyme B G/G or A/G genotype on the reduced TRM in patients with myeloid malignancies might be a consequence of increased resistance to infections in these recipients. This hypothesis, although attractive, is highly speculative and is not supported by the present study because of the unavailability of data on the causes of infections in this cohort. Further studies will be needed to clarify whether the granzyme B genotypes can differentially affect the responses of patients against infections.

Two recent reports have described a significant correlation between disease susceptibility and the RAH/QPY polymorphism in the granzyme B gene. The wild type QPY genotype was associated with an increased incidence of Epstein-Barr-virus-associated hemophagocytic lymphohistiocytosis (HLH) in children.[32] Conversely, a subsequent study reported an association of the mutant RAH genotype with the incidence of breast cancer.[33] However, to link the genetic susceptibility of granzyme B to disease based on the presented data is difficult, because no patient developed HLH or breast cancer following HSCT in the current cohort.

In conclusion, the present data suggest that the granzyme B polymorphism may affect the prognosis after BMT from an unrelated donor, and therefore, the granzyme B genotyping in transplant donors and recipients may provide opportunities to choose an ideal donor. However, care should be made in drawing conclusions, because the number of patients evaluated in the present study is limited. Experimental evidence is also required to substantiate the effects of extracellular granzyme B according to the polymorphism on organ and tissue damage. Further studies are warranted to ascertain whether the findings of this study can be extended to other disease groups, other stem cell sources, or HLA-mismatched transplantation, as well as to validate the present data.
